# Early-Life Oat Supplementation Is Associated with Altered Cecal Microbiota and Microbial Metabolic Profiles and Reduced *Campylobacter jejuni* Colonization in Broiler Chickens

**DOI:** 10.3390/ijms27198607

**Published:** 2026-09-26

**Authors:** Bereket Dessalegn, Motuma Debelo, Nicola Palmieri, Károly Dublecz, Michael Hess, Wageha A. Awad

**Affiliations:** 1 Clinical Unit for Poultry Medicine, Clinical Department for Farm Animals and Food System Transformation, University of Veterinary Medicine, 1210 Vienna, Austria; bereket.awoke@vetmeduni.ac.at (B.D.); motuma.dibaba@vetmeduni.ac.at (M.D.); palmierin@staff.vetmeduni.ac.at (N.P.); michael.hess@histovac.com (M.H.); 2 Institute of Physiology and Nutrition, Georgikon Campus, Hungarian University of Agriculture and Life Science, 8360 Keszthely, Hungary; dublecz.karoly@uni-mate.hu

**Keywords:** broiler chickens, *Campylobacter jejuni*, cecal microbiota, oat, shotgun metagenomics, short-chain fatty acids, microbial metabolism, food safety

## Abstract

*Campylobacter jejuni* is a leading foodborne pathogen that commonly colonizes the intestinal tract of broiler chickens and represents a significant food safety concern. Dietary intervention may provide a practical approach to modulate the gut microbiota and reduce pathogen colonization. This study investigated the effects of oat supplementation on *C. jejuni* colonization and dissemination, cecal microbiota and predicted functions, and cecal short-chain fatty acids (SCFAs) and ileal amino acid profiles during early-life infection in broiler chickens. One-day-old Ross 308 broiler chickens were fed a basal diet or diets containing 10% oat (MDF) or 20% oat (HDF) and orally infected with *C. jejuni* at 2 days of age. *C. jejuni* loads in the cecum, jejunum, liver, and spleen were quantified by culture. Cecal microbiota composition and predicted functional profiles were characterized by shotgun metagenomic sequencing, while cecal SCFAs and ileal amino acids were quantified at 21 and 35 dpi using gas chromatography and an automatic amino acid analyzer, respectively. Oat supplementation reduced intestinal *C. jejuni* loads and limited dissemination to the liver and spleen, with the MDF diet reducing cecal loads by up to 3.3 log_10_ CFU/g at 35 dpi (*p* < 0.05). Infection altered cecal microbial diversity and community structure, while oat supplementation modified these infection-associated microbial patterns, with MDF showing the most consistent effects. Differential abundance analysis identified changes in taxa, including *Bacteroides*, *Parabacteroides*, and *Phocaeicola*, together with changes in predicted functions related to motility and chemotaxis, iron acquisition, and dormancy/sporulation. These microbial changes coincided with alterations in cecal isobutyrate and ileal amino acids, particularly glutamate, serine, proline, and aspartate, in MDF-fed infected chickens. Overall, MDF supplementation was most consistently associated with reduced *C. jejuni* colonization and dissemination and concurrent changes in the developing cecal microbial and metabolic profiles.

## 1. Introduction

*Campylobacter jejuni* (*C. jejuni*) is a major zoonotic pathogen and one of the leading causes of bacterial gastroenteritis worldwide [1]. Human campylobacteriosis commonly presents with diarrhea, often accompanied by fever, abdominal pain, nausea, and vomiting. Most infections are self-limiting; however, severe or invasive disease can occur, and post-infectious complications, including Guillain-Barre syndrome and reactive arthritis, have been reported [2]. *C. jejuni* is therefore an important foodborne pathogen with substantial public health implications [3,4]. Broiler chickens are a major reservoir of *C. jejuni* and an important source of human exposure through contaminated poultry products and cross-contamination during food handling [5,6]. Unlike humans, broiler chickens can harbor high intestinal loads of *C. jejuni*, particularly in the ceca, without apparent clinical signs, allowing infected flocks to serve as important reservoirs for transmission along the poultry production chain [7].

Reducing *C. jejuni* colonization in the broiler intestine before slaughter is an important preharvest strategy for limiting carcass contamination and subsequent human exposure [8]. However, interventions targeting *Campylobacter* colonization in poultry, including biosecurity measures, feed and water additives, probiotics, bacteriophages, and vaccinations, have shown variable and often inconsistent efficacy under experimental and field conditions [9]. Consequently, increasing attention has been directed toward nutritional strategies that target the intestinal microbial ecosystem. Dietary interventions that modify microbial community composition and metabolic activity have therefore been investigated as potential approaches for limiting *C. jejuni* colonization in broiler chickens [10,11,12].

Interestingly, *C. jejuni* is rarely detected in broiler chicks during the first 1–3 weeks of life, with flock-level colonization becoming more prevalent after the third week and increasing toward market age [13]. Nevertheless, experimental studies show that young chicks can be colonized and shed *Campylobacter* [14], whereas evidence for early flock-level colonization under commercial conditions remains limited [15]. This age-dependent pattern suggests that the early post-hatch gastrointestinal environment may be less permissive to *Campylobacter* establishment, although the underlying mechanisms remain incompletely understood. Maternally derived antibodies may contribute to this protection, but their levels and protective effects decline with age [13,16]. The intestinal microbiota may also contribute through microbial competition, nutrient utilization, metabolite production, and interactions with the intestinal environment [17,18,19]. Thus, factors shaping microbial development during this period may influence susceptibility to *C. jejuni* colonization.

Given the rapid development of the intestinal microbiota after hatch, nutritional factors that alter microbial substrate availability and fermentation may influence the establishment of colonization resistance [20]. Dietary fiber (DF) has therefore emerged as a promising nutritional strategy for modulating intestinal microbial ecology and supporting gut health in poultry [21,22]. DF comprises diverse non-starch polysaccharides (NSPs), oligosaccharides, and lignin with distinct physicochemical and fermentative properties [21]. Although largely resistant to host digestion, fermentable DF components can be metabolized by intestinal microorganisms, producing monosaccharides and fermentation products, including SCFAs [23]. SCFAs can influence luminal pH, epithelial barrier function, immune responses, and microbial community composition, with effects depending on the specific metabolite, concentration, site of production, and physiological context [24,25]. Consistent with these functions, several dietary interventions targeting fiber availability and the intestinal microbiota have been associated with reduced *C. jejuni* colonization in broiler chickens [7,26,27,28,29].

Despite these promising findings, the biological effects of DF depend on its source, composition, physicochemical properties, fermentability, and inclusion level [30]. These characteristics can differentially influence intestinal microbial ecology, metabolite production, digesta properties, nutrient utilization, and host physiology [24,31]. Oat is a distinctive cereal feed ingredient containing both soluble fiber, particularly β-glucan, and insoluble structural fiber fractions, making it a relevant source of DF for poultry nutrition [32,33]. These properties can affect digesta viscosity, microbial fermentation, and gut microbial community structure [34]. Recent studies in broiler chickens have shown that whole oat or dehulled oat diets can modify cecal microbial composition and SCFA profiles, supporting the potential of oat to modulate the intestinal microbial ecosystem [32,35,36]. Importantly, the inclusion level of oat can differentially influence microbial composition, fermentation activity, and host responses, highlighting the need to optimize oat inclusion levels to achieve desirable physiological and microbial outcomes [37,38].

Although oat-based diets have been associated with improved intestinal health and reduced *C. jejuni* colonization in broiler chickens, the cascade how early-life oat supplementation relates to the development of the intestinal microbial ecosystem and its metabolic activity during *C. jejuni* exposure remains insufficiently characterized. Previous unpublished experimental work from our group investigated a similar dietary intervention in broiler chickens infected with *C. jejuni* at 14 days of age and provided preliminary evidence of effects on cecal microbiota, intestinal barrier-related responses and cecal *C. jejuni* colonization. The present study extends this work in several important respects. First, *C. jejuni* exposure was initiated at 2 days of age, during an early stage of post-hatch intestinal development. Second, beyond quantifying cecal colonization, viable *C. jejuni* was quantified in the jejunum, liver and spleen to examine intestinal and extraintestinal dissemination. Third, we characterized cecal microbial diversity and composition together with microbial metabolic profiles to investigate whether early-life oat supplementation was associated with concurrent changes in the microbial ecosystem and its functional output. Thus, the present study provides a broader assessment of the associations among early-life dietary intervention, *C. jejuni* colonization and dissemination, and host-associated microbial and metabolic responses.

Accordingly, this study aimed to investigate the associations of early-life oat supplementation with *C. jejuni* colonization and dissemination, cecal microbiota composition and functional potential, and cecal short-chain fatty acid and ileal amino acid profiles in broiler chickens infected at 2 days of age.

## 2. Results

### 2.1. Effect of Oat Supplementation on Intestinal Campylobacter jejuni Colonization and Systemic Dissemination

No mortality occurred in any treatment group during the experimental period. Oat supplementation reduced *C. jejuni* colonization in the intestine and its recovery from extraintestinal tissues, with effects becoming evident from 21 and 14 dpi, respectively (Figure 1). Chickens fed the MDF diet had significantly lower cecal *C. jejuni* loads than infected chickens fed the basal diet from 21 dpi onwards, with reductions of approximately 2.4, 1.3, and 3.3 log_10_ CFU/g at 21, 28, and 35 dpi, respectively (*p* < 0.05). Similarly, both MDF and HDF significantly reduced jejunal *C. jejuni* loads, although the reductions were consistently greater with MDF, reaching approximately 1.6 log_10_ CFU/g at 35 dpi. In extraintestinal tissues, oat supplementation reduced *C. jejuni* recovery from the liver and spleen at 14 and 21 dpi, with reductions ranging from approximately 1.0 to 1.5 log_10_ CFU/g compared with infected chickens fed a basal diet (*p* < 0.05). Notably, *C. jejuni* was not detected in the liver or spleen of oat-fed chickens at 28 and 35 dpi.

### 2.2. Effects of Oat Supplementation and Campylobacter jejuni Infection on the Cecal Microbiota

#### 2.2.1. Alpha Diversity of the Cecal Microbiota

Cecal microbiota alpha diversity, assessed using the Shannon diversity index, varied with *C. jejuni* infection and dietary treatment across sampling time points (Figure 2). At 14 dpi, neither diet nor infection significantly affected Shannon diversity, and no significant diet × infection interaction was detected (*p* ≥ 0.277). At 21 dpi, *C. jejuni* infection significantly affected Shannon diversity (*p* < 0.001), with infected chickens showing higher Shannon diversity than non-infected chickens, whereas neither the main effect of diet nor the diet × infection interaction was significant (*p* = 0.421 and *p* = 0.606, respectively). At 28 dpi, significant effects of diet (*p* = 0.009) and infection (*p* < 0.001), as well as a significant diet × infection interaction (*p* = 0.024), were observed. At 35 dpi, infection remained significant (*p* < 0.001), and the diet × infection interaction was also significant (*p* = 0.036), whereas the main effect of diet was not significant (*p* = 0.521). Thus, the effects of oat supplementation on Shannon diversity differed according to infection status at 28 and 35 dpi, with distinct patterns observed between the MDF and HDF diets.

#### 2.2.2. Beta Diversity and Community Structure of the Cecal Microbiota

Bray–Curtis-based NMDS analysis revealed differences in cecal microbial community structure between *C. jejuni*-infected and non-infected chickens across experimental groups (Figure 3). At 14 dpi, microbial communities showed relatively limited separation between infected groups, whereas greater separation became evident at 21 dpi. PERMANOVA identified *C. jejuni* infection as a significant driver of community structure at 21 dpi (*R*^2^ = 0.21, *p* = 0.001). The separation between infected and non-infected groups decreased at 28 dpi, followed by partial separation at 35 dpi. At 28 dpi, the MDF treatment was associated with a significant difference in cecal microbial community structure relative to the infected basal diet group (*R*^2^ = 0.05, *p* = 0.011), with MDF-treated communities showing greater overlap with those of non-infected controls in the NMDS ordination.

#### 2.2.3. Differential Abundant Bacterial Species

Age-stratified MaAsLin3 analysis identified 24 bacterial species exhibiting significant diet × infection interactions (*q* < 0.1) at two or more sampling time points (Figure 4). These comprised 11 *Bacteroides* species, six *Parabacteroides* species, three *Phocaeicola* species, and one species each from *Barnesiella*, *Butyricimonas*, *Ligilactobacillus*, and *Tannerella*. Significant interactions were predominantly detected at 28 and 35 dpi, with 22 significant species identified at each time point, compared with nine at 14 dpi and one at 21 dpi. Of the 24 species, 20 showed significant diet × infection interactions at both 28 and 35 dpi, whereas six species (*Bacteroides stercoris*, *Butyricimonas faecalis*, *Ligilactobacillus salivarius*, *Parabacteroides goldsteinii*, *Parabacteroides johnsonii*, and *Tannerella forsythia*) showed significant interactions at three sampling time points. Pattern classification showed that nearly all significant interactions followed a Rescue pattern, characterized by a shift in the oat-supplemented groups toward the abundance observed in the corresponding non-infected controls. *Ligilactobacillus salivarius* at 28 dpi was the only species classified as a Mixed pattern, and no taxa exhibited a Divergent pattern.

#### 2.2.4. Predicted Functional Profiles of the Cecal Microbiota

Predicted microbial functional profiles based on SEED subsystem annotations showed age- and treatment-dependent patterns across the experimental period (Figure 5). In chickens fed the basal diet, *C. jejuni* infection was associated with higher relative abundance scores for functional categories related to dormancy and sporulation, mobile genetic elements, motility and chemotaxis, and iron acquisition, particularly at 21 dpi. The patterns observed with oat supplementation varied according to sampling age and functional category rather than following a uniform response. At 21 and 28 dpi, the HDF group showed more pronounced differences in the standardized scores of categories related to secondary metabolism, motility and chemotaxis, dormancy and sporulation, and mobile genetic elements. In contrast, at 35 dpi, the MDF group showed more pronounced differences in the standardized scores for secondary metabolism.

### 2.3. Cecal SCFA Concentrations

Cecal SCFA concentrations showed treatment- and time-dependent patterns across the experimental period (Figure 6). Among the SCFAs analyzed, only isobutyrate showed a significant diet × infection interaction, with higher concentrations observed at 35 dpi in *C. jejuni*-infected chickens receiving MDF than in infected chickens fed the basal diet (*p* < 0.05). No significant diet × infection interactions were detected for acetate, propionate, butyrate, isovalerate, or valerate at either sampling time point (*p* > 0.05). Although butyrate concentrations were numerically higher in *C. jejuni*-infected chickens receiving oat supplementation than in infected chickens fed the basal diet, these differences were not statistically significant.

### 2.4. Ileal Amino Acid Profiles

Ileal amino acid profiles were significantly influenced by the diet × infection interaction at both sampling time points (Figure 7A). At 21 dpi, significant interactions were detected for arginine, glycine, leucine, methionine, phenylalanine, proline, serine, and valine (*p* < 0.05). At 35 dpi, significant interactions were observed for 15 of the 17 amino acids analyzed, with leucine and methionine showing no significant interaction. Among the amino acids associated with *C. jejuni* metabolism, infection was associated with lower concentrations of glutamate, serine, and proline at 21 dpi (*p* < 0.05). At 35 dpi, *C. jejuni-infected* chickens fed the basal diet had significantly lower concentrations of serine, glutamate, proline, and aspartate than non-infected controls (*p* < 0.05), whereas these differences were not observed in the oat-supplemented groups, particularly the MDF group. Principal component analysis (PCA) further showed separation between non-infected and *C. jejuni*-infected chickens fed the basal diet at 21 dpi, whereas greater overlap was observed between infected and non-infected chickens receiving oat supplementation. At 35 dpi, the six diet × infection groups formed distinct clusters, indicating greater divergence in the overall ileal amino acid profiles among treatment groups (Figure 7B).

## 3. Discussion

Campylobacteriosis remains a major public health concern worldwide [3,4]. Increasing evidence suggests that diet-driven modulation of the gut microbiota and its metabolic activity may influence *C. jejuni* colonization and persistence within the chicken gastrointestinal tract [28]. In the present study, oat supplementation was associated with reduced intestinal *C. jejuni* colonization and recovery from extraintestinal tissues, together with changes in cecal microbial diversity, community composition, and predicted functional potential in broiler chickens. These responses were age-dependent and accompanied by selective changes in cecal SCFA concentrations and ileal amino acid profiles. The magnitude and timing of these responses differed between the MDF and HDF diets, with MDF showing the most consistent associations with reduced *C. jejuni* colonization and microbial community changes. Collectively, these findings indicate an association between early-life oat supplementation and coordinated change in the developing cecal microbial ecosystem and metabolic profiles that coincided with reduced *C. jejuni* colonization and dissemination; however, the underlying mechanisms remain to be established.

Interestingly, the present study employed an early-life *C. jejuni* infection model, with chickens inoculated at 2 days of age during an active period of gut microbiota establishment and immune maturation. Although young broiler chickens are generally less susceptible to *C. jejuni* colonization during the first weeks of life [13,14], experimental exposure during this period can modify the developing intestinal microbial community [19]. In this context, the observed differences associated with oat supplementation reflects the influence of dietary fiber on host–microbiota–pathogen interactions during early gut development, when microbial community assembly and colonization resistance are still developing. These findings highlight early-life dietary intervention as a potential approach for modulating the intestinal environment during the period of *C. jejuni* exposure.

Oat supplementation was associated with reduced *C. jejuni* colonization in both the cecum and jejunum compared with the infected basal diet, with the greatest reduction in cecal counts reaching approximately 3.3 log_10_ CFU/g at 35 dpi. These findings are consistent with previous studies demonstrating that dietary modulation of the intestinal environment and microbiota can influence *C. jejuni* colonization in poultry. Dietary interventions including exogenous enzyme cocktails, phytogenic feed additives, and fermented rapeseed meal have been reported to reduce intestinal *C. jejuni* loads and modify the intestinal microbial community in broiler chickens [7,24,26,28]. Beyond intestinal colonization, previous studies have demonstrated that *C. jejuni* infection can compromise intestinal barrier function and facilitate the translocation of enteric microorganisms to extraintestinal tissue [39], while dietary interventions targeting intestinal function and the gut microbiota have been associated with reduced recovery of *C. jejuni* from extraintestinal organs [40]. In the present study, the relatively high recovery of *C. jejuni* from the liver and spleen at the early sampling point may reflect systemic dissemination following oral inoculation at 2 days of age, when the intestinal barrier, immune system, and gut microbiota were still developing. Bacterial recovery subsequently declined, while oat supplementation was associated with an additional reduction of approximately 1.0–1.5 log_10_ CFU/g in the liver and spleen and with no detectable *C. jejuni* at 28 and 35 dpi, suggesting that its effect extended beyond intestinal colonization to reduced detection of the pathogen in extraintestinal tissues.

The responses to oat supplementation were generally more pronounced with MDF than HDF, particularly for cecal *C. jejuni* colonization and microbiota-associated responses. The impact of dietary fiber can depend on both its source and inclusion level, with increasing fiber concentration influencing digesta characteristics, nutrient utilization, microbial fermentation, and gut microbial composition in ways that are not necessarily linear [30,37]. Thus, the higher oat inclusion level may have generated a different physicochemical and fermentative environment from that produced by MDF. The concurrent changes in microbial community structure, predicted functional profiles, and selected metabolites observed in the present study are consistent with this possibility. However, because the physicochemical properties of digesta and fermentation kinetics were not directly assessed, the mechanisms underlying the greater response to MDF remain uncertain.

Early-life *C. jejuni* infection was associated with temporal changes in cecal microbial alpha diversity. In the present study, *C. jejuni* infection increased Shannon diversity in chickens fed the basal diet. Although higher microbiota diversity is often considered indicative of healthy microbiota, the increase observed here may instead reflect infection-associated community restructuring rather than improved microbial stability. In agreement with this interpretation, previous studies in chickens have reported that *C. jejuni* infection did not reduce microbial diversity despite marked alterations in community composition [41]. Because the Shannon index integrates species richness and evenness, changes in either component can increase or decrease the index without necessarily indicating improved or impaired microbial health [42]. Oat supplementation further modified infection-associated alpha diversity patterns, with significant diet × infection interactions observed at 28 and 35 dpi, indicating that the effect of oat supplementation on Shannon diversity depended on infection status. The direction and magnitude of these responses differed between MDF and HDF, suggesting that dietary inclusion level influenced the microbial response to *C. jejuni* infection. Similar effects of dietary fiber supplementation on microbial diversity have been reported in broiler chickens following lignocellulose [43] and moderate levels of arabinoxylan [22]. Thus, the observed changes in alpha diversity should be interpreted as alterations in the ecological response of the cecal microbiota to infection and dietary intervention, rather than as evidence of improved microbiome health or resilience.

Cecal microbial community structure differed significantly between *C. jejuni*-infected and non-infected broiler chickens, with the greatest separation observed at 21 dpi. These findings are consistent with previous reports demonstrating that *C. jejuni* colonization can alter cecal beta diversity and overall microbial community structure [44,45], indicating substantial infection-associated changes in the intestinal microbial ecosystem. Beyond the effect of infection, oat supplementation was associated with distinct patterns of cecal microbial community structure, particularly in MDF-supplemented chickens [21,22]. Notably, at 28 dpi, MDF supplementation shifted the microbial community structure of infected chickens toward that of non-infected controls, suggesting attenuation of infection-associated community changes at this time point. This pattern indicates that the effect of dietary oat extended beyond changes in alpha diversity to broader alterations in community composition. Such restructuring may reflect changes in the ecological conditions of the cecum and could influence microbial interactions and substrate utilization; however, these potential mechanisms were not directly assessed in the present study.

Differential abundance analysis further demonstrated that oat supplementation was associated with changes in several bacterial taxa, particularly species belonging to the genera *Bacteroides*, *Parabacteroides*, and *Phocaeicola*, with many of these taxa exhibiting a rescue pattern in response to *C. jejuni* infection. These findings are consistent with previous studies reporting changes in the abundance of *Bacteroides* and *Parabacteroides* following dietary fiber supplementation [43,46], including supplementation with β-glucan and arabinoxylan [22]. Members of the genus *Bacteroides* possess extensive capacities for degrading complex dietary polysaccharides within the intestinal environment [47,48]. The concurrent changes in fiber-responsive taxa, predicted microbial functional profiles, and selected metabolites observed in the present study indicate that oat supplementation was associated with broad changes in the cecal microbial ecosystem. These microbial changes occurred alongside reduced *C. jejuni* colonization, although the present study cannot establish whether specific bacterial taxa or their associated functions directly mitigated this reduction.

Beyond changes in microbial composition and community structure, oat supplementation was associated with age-dependent differences in the predicted functional profiles of the cecal microbiota. In chickens fed the basal diet, *C. jejuni* infection was associated with higher relative abundances of functional categories related to motility and chemotaxis, dormancy and sporulation, mobile genetic elements, and iron acquisition, particularly at 21 dpi, whereas the patterns observed in oat-supplemented groups varied according to dietary inclusion levels and sampling age. These functional categories are broadly involved in bacterial adaptation, environmental sensing, survival, and nutrient acquisition and may therefore be relevant to microbial responses to the intestinal environment [49,50]. Together with the observed changes in bacterial taxa, these findings suggest that oat supplementation was associated with changes in the predicted functional profile of the developing cecal microbiota during *C. jejuni* infection. Whether these functional changes contributed to the reduced pathogen colonization observed in oat-supplemented chickens remains to be determined.

Oat supplementation had limited effects on the overall cecal SCFA profile; MDF was associated with higher isobutyrate concentrations in *C. jejuni*-infected chickens at 35 dpi. Similar associations between isobutyrate concentrations and infection-related phenotypes have been reported in chickens [51]. Isobutyrate, a branched-chain fatty acid derived from microbial amino acid fermentation, can also serve as an energy substrate for intestinal epithelial cells, although its contribution appears to be less prominent than that of butyrate [52]. However, the biological significance of the increased isobutyrate observed in the present study remains uncertain. The increase coincided with reduced *C. jejuni* colonization in MDF-fed chickens, but whether isobutyrate directly contributed to pathogen inhibition was not determined.

Amino acid availability represents another factor potentially influencing *C. jejuni* colonization dynamics, as this pathogen can utilize amino acids, including aspartate, glutamate, proline, and serine, as primary carbon and energy sources [53]. In the present study, *C. jejuni* infection was associated with reduced concentrations of these amino acids in chickens receiving the basal diet, consistent with previous reports of amino acid depletion during intestinal colonization [54]. In contrast, MDF supplementation was associated with higher concentrations of aspartate, glutamate, proline, and serine in infected chickens. This pattern may reflect the lower intestinal *C. jejuni* burden in oat-supplemented chickens and, consequently, reduced pathogen-associated utilization of these substrates. Alternatively, changes in the gut microbiota may have influenced amino acid availability through altered nutrient cycling, microbial cross-feeding, and amino acid turnover [55]. These findings indicate that oat supplementation was associated with concurrent changes in microbial composition, predicted functional profiles, and intestinal metabolites during *C. jejuni* infection. Whether these metabolic changes contribute directly to reduced pathogen colonization requires further investigation.

Several limitations should be considered when interpreting these findings. Unfortunately, the protein content of the experimental diets was not identical, with the control diet containing slightly more protein than the MDF and HDF diets. However, several responses were more pronounced in chickens receiving the MDF diets, despite the protein contents of the MDF and HDF diets being similar. This suggests that the differences in dietary protein may have had a limited contribution to the observed treatment responses. The differences in dietary protein may have contributed to the observed differences in ileal amino acid profiles; however, assuming an average protein digestibility of 80%, a 1% difference in dietary protein would correspond to approximately 0.2% undigested protein reaching the ileum. Moreover, ileal amino acid concentrations are influenced by several factors, including gizzard function, pancreatic enzyme secretion, intestinal absorption, and mucosal turnover. Therefore, the differences in dietary protein should be considered a potential confounding factor when interpreting the observed responses. Although oat supplementation was associated with concurrent changes in *C. jejuni* colonization, microbial composition, predicted functional profiles, and selected metabolites, the study was not designed to establish a direct causal relationship between microbiome changes and reduced pathogen colonization. Shotgun metagenomic profiling provides information on the functional potential of the microbial community but does not directly measure gene expression or metabolic activity, while SCFA and amino acid analyses capture only selected aspects of microbial and host-associated metabolism. In addition, the controlled experimental conditions, use of Ross 308 broilers, and experimental *C. jejuni* infection may limit the generalizability of these findings to naturally infected chickens and commercial production systems. Future studies integrating broader metabolomic and transcriptomic approaches, together with experimental manipulation of the microbiome, could help clarify the mechanisms underlying the association between dietary oat supplementation, microbial metabolism, and *C. jejuni* colonization.

## 4. Materials and Methods

### 4.1. Experimental Design and Sample Collection

A total of 180 one-day-old mixed sex Ross-308 broiler chickens were obtained from a commercial hatchery and randomly assigned to six experimental treatment groups (n = 30 chickens/group), with five replicate pens per treatment and six chickens per pen. Each treatment was housed in a separate experimental room at the Clinic for Poultry Medicine, University of Veterinary Medicine Vienna, with infected and non-infected treatments maintained in physically separate rooms to minimize cross-contamination. Chickens were reared on wood shaving litter and provided feed and water ad libitum throughout the experimental period. The experimental diets were formulated with oat inclusion levels designed to provide moderate (MDF) and high (HDF) dietary fiber concentrations while maintaining comparable metabolizable energy and nitrogen concentrations across treatment groups. The lower energy content of oats was compensated for by increased oil supplementation. Consequently, the oat-containing diets had higher fiber and crude fat contents and lower starch concentrations than the control diet (Table 1). The diets were formulated to meet the nutritional requirements of Ross-308 broiler chickens [56]. Although some differences in the crude protein and amino acid composition remained among all diets, all nutrients, including essential amino acids, met or exceeded the nutritional requirements of the broiler chickens. The six experimental groups were: (1) basal diet (control); (2) basal diet containing 10% oat (MDF); (3) basal diet containing 20% oat (HDF); (4) basal diet + *C. jejuni*; (5) MDF + *C. jejuni*; and (6) HDF + *C. jejuni*. On day 1, cloacal swabs were collected from all chickens and cultured on modified charcoal cefoperazone deoxycholate agar (mCCDA) to confirm the absence of *Campylobacter* spp. before experimental inoculation. On day 2, chickens in groups 4–6 were orally inoculated by crop gavage with *C. jejuni* (NCTC 12744) at a dose of 1 × 10^8^ CFU/chicken, as previously described [57]. Chickens were monitored daily throughout the experimental period for clinical signs, general health, and mortality.

At 14, 21, 28, and 35 days post-infection (dpi), five chickens from each treatment group were randomly selected, with one chicken sampled from each replicate pen, and euthanized by intravenous administration of thiopental (20 mg/kg body weight) via the wing vein, followed by exsanguination through jugular vein bleeding. Cecal and jejunal contents and liver and spleen tissues were aseptically collected for *C. jejuni* quantification by culture. Cecal contents were additionally collected for microbiota profiling and short-chain fatty acids (SCFA) analysis, whereas ileal contents were collected for amino acid profiling. All samples were immediately frozen and stored at −80 °C until further processing.

### 4.2. Enumeration of Campylobacter jejuni

*Campylobacter jejuni* loads in the cecum, jejunum, liver and spleen were quantified by plating tenfold serial dilutions of homogenized samples prepared in phosphate-buffered saline (PBS) onto *Campylobacter* Selective Agar (CASA, bioMerieux Austria GmbH, Vienna, Austria) in duplicate. Plates were incubated under microaerophilic conditions at 41.5 °C for 48 h. Bacterial counts were calculated based on colony counts and corresponding dilution factors and expressed as log_10_ colony-forming units per gram (log_10_ CFU/g) of sample.

### 4.3. DNA Extraction and Library Preparation

Genomic DNA was extracted from cecal contents using the MagBind^®^ Environmental DNA 96 Kit (Omega Bio-Tek, Norcross, GA, USA) in combination with the KingFisher automated purification system (Thermo Fisher Scientific, Waltham, MA, USA). DNA quality was assessed by gel electrophoresis, and DNA concentration was quantified using a Qubit 3.0 fluorometer (Thermo-Fischer Scientific, Waltham, MA, USA). Sequencing libraries were prepared using the Illumina Nextera library preparation kit according to an in-house optimized protocol (Illumina, San Diego, CA, USA).

### 4.4. Shotgun Metagenomic Sequencing and Bioinformatics Analysis

Paired-end shotgun metagenomic sequencing (2 × 150 bp) was performed on an Illumina NextSeq 500 platform. For the cecal samples analyzed in the present study, sequencing generated 3,318,908,830 raw reads. Raw reads were processed using the Sunbeam pipeline. Initial sequence quality was assessed using FastQC v0.11.5 [58]. Reads were preprocessed using fastp v0.24.0 [59] for adapter removal, quality trimming, low-complexity filtering, and removal of reads shorter than 36 bp. Host-derived reads were removed using Hostile v1.1.0 [60] by alignment against the *Gallus gallus* reference genome (GRCg6a). Following preprocessing and host-read removal, 3,079,637,384 reads were retained for downstream analysis, corresponding to 92.8% of the raw reads. The median number of retained reads per cecal sample was 20.23 million. Taxonomic classification was performed using Kraken2 with the PlusPF database (release date: 5 June 2024). Functional profiling was performed using Super-Focus v4 [61], with filtered reads mapped to the SEED database using translated homology searches and annotated according to Subsystem functional levels 1–3.

### 4.5. Determination of Metabolites

Short-chain fatty acid (SCFAs) concentrations were determined by gas chromatography (TRACE 2000, Thermo Fisher Scientific, Waltham, MA, USA) as previously described [62]. Briefly, cecal contents were thawed and homogenized, after which 250 μL of digesta was acidified with 600 μL of 1.11 M HCl. Samples were analyzed using a gas chromatograph equipped with a 30 m fused-silica capillary column (0.25 mm internal diameter; Nukol, Supelco Inc., Bellefonte, PA, USA) and a flame-ionization detector (FID) with a split injector ratio of 1:50. The injection volume was 1 μL at 220 °C, and detection was performed at 250 °C. Helium was used as the carrier gas at a pressure of 83 kPa. External calibration curves were generated using SCFA standard mixtures containing acetate, propionate, n-butyrate and n-valerate at concentrations of 1, 4, 8 and 20 mM, respectively. In addition, amino acid concentrations in ileal contents were determined using an automatic amino acid analyzer (Ingos Amino Acid Analyser AAA 400, INGOS, Prague, Czech Republic), as previously described [63].

### 4.6. Statistical Analysis

Statistical analyses of *C. jejuni* intestinal colonization and systemic dissemination were performed using GraphPad Prism (v10.6; GraphPad Software, San Diego, CA, USA). Bacterial counts are presented as mean ± standard error (SE), expressed as log_10_ CFU/g. These outcomes were analyzed by two-way ANOVA with diet and time as fixed effects, followed by Tukey’s post hoc test. SCFA and ileal amino acid concentrations were analyzed in R (v4.3). At each sampling time point (21 and 35 dpi), each metabolite was analyzed separately using Type III two-way ANOVA with diet, infection status, and their interaction as fixed effects, applying sum-to-zero contrasts. Statistical significance was set at *p* < 0.05 unless otherwise stated, and data are presented as mean ± standard error (SE). Principal component analysis (PCA) of the ileal amino acid profiles was performed after centering and scaling the concentrations of all 17 amino acids using the prcomp function in R. PCA was fitted across all samples to generate common principal component axes, and sample scores were visualized according to diet × infection group within each sampling time point.

Microbiome analyses were performed in R (v4.3). Alpha diversity was assessed using the Shannon diversity index. Differences were analyzed by Type III two-way ANOVA with diet, infection status, and time as fixed effects, followed by Tukey’s post hoc test. Beta diversity based on Bray–Curtis dissimilarity was evaluated using permutational multivariate analysis of variance (PERMANOVA; ADONIS), using diet, infection status, time, and their interactions as explanatory variables, and visualized by non-metric multidimensional scaling (NMDS).

Differential abundance of cecal bacterial species was assessed using MaAsLin3 in R. Separate models were fitted for each sampling time point (14, 21, 28, and 35 dpi), with species abundance modeled as a function of diet, infection status, and their interaction (diet × infection), using the basal diet and uninfected chickens as the reference levels. Species abundances were normalized by total-sum scaling and log-transformed. Species were retained if they reached a relative abundance of at least 0.01% in ≥20% of samples at the corresponding sampling time point. Multiple testing was controlled using the Benjamini–Hochberg false discovery rate, and diet × infection interactions were considered significant at *q* < 0.1.

Significant interactions were classified according to the direction of the diet × infection effect. An oat diet was considered to attenuate the infection-associated difference when the interaction coefficient was significant (*p* < 0.05) and the absolute infected-uninfected difference was smaller than that of the basal diet. Conversely, it was considered to enhance the infection-associated difference when the interaction coefficient was significant (*p* < 0.05) and the absolute infected-uninfected difference exceeded that of the basal diet. Interaction patterns were classified as Rescue when at least one oat diet attenuated the infection-associated difference and neither enhanced it, Divergent when at least one enhanced the difference and neither attenuated it, and Mixed when the two oat diets produced opposing patterns [64].

## 5. Conclusions

This study demonstrates that early-life oat supplementation was associated with reduced *C. jejuni* colonization in the cecum and jejunum resulting in limited dissemination to the liver and spleen in broiler chickens infected at 2 days of age. The 10% oat (MDF) diet showed the most consistent effects, coinciding with changes in cecal bacterial groups, particularly *Bacteroides*, *Parabacteroides*, and *Phocaeicola*, and predicted microbial functions related to motility and chemotaxis, iron acquisition, and dormancy/sporulation. Oat supplementation also altered cecal isobutyrate and ileal amino acid profiles, including glutamate, serine, proline, and aspartate. These findings support MDF as a promising nutritional strategy for modulating the intestinal microbial environment and reducing *C. jejuni* colonization. Future mechanistic studies are needed to determine the contribution of these microbial and metabolic changes to pathogen reduction and their relevance to poultry food safety and One Health.

## Figures and Tables

**Figure 1:**
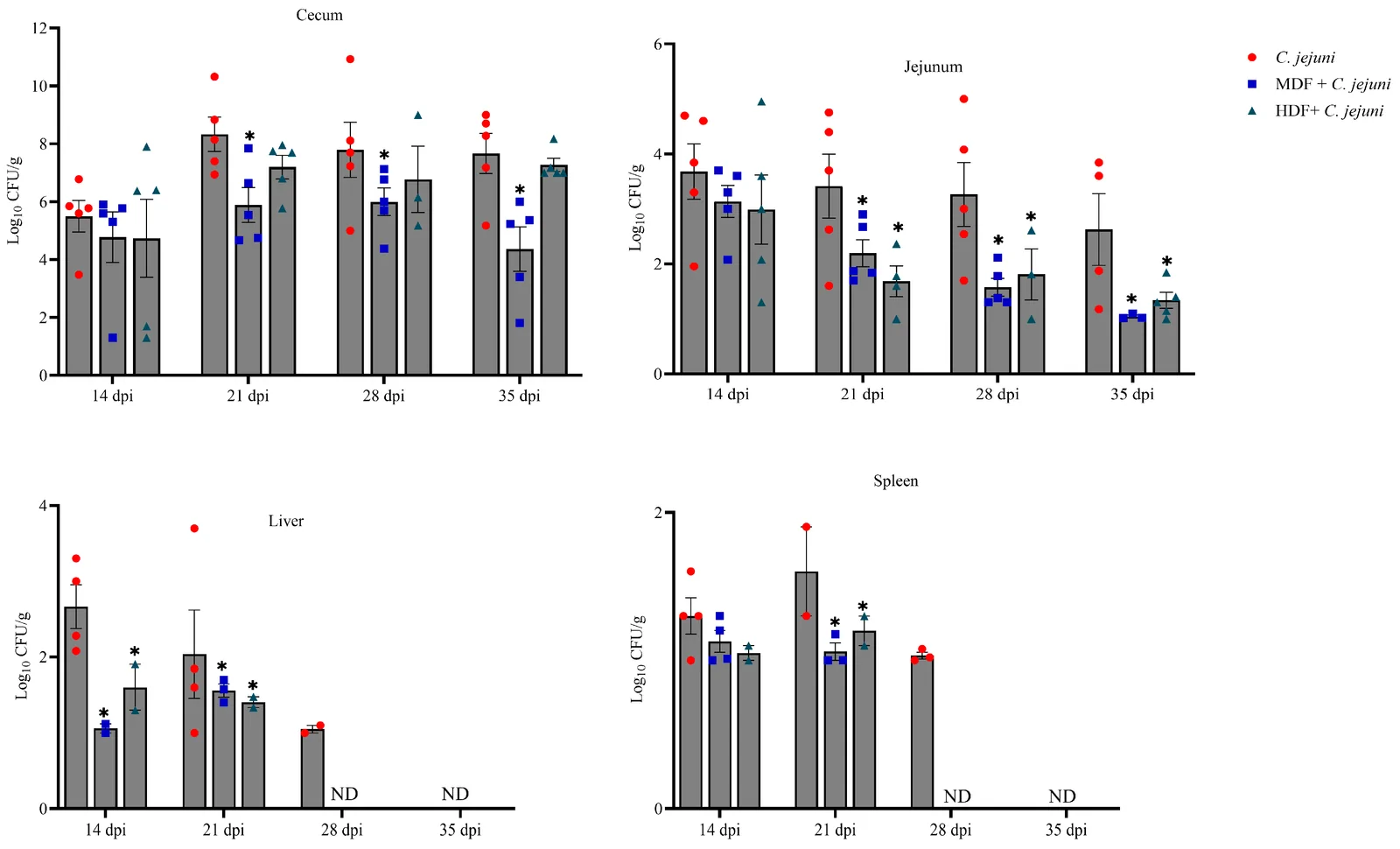
*C. jejuni* counts in the cecum, jejunum, liver, and spleen of broiler chickens from the three *C. jejuni*-infected groups: basal diet + *C. jejuni*, MDF + *C. jejuni*, and HDF + *C. jejuni* (groups 4–6) at 14, 21, 28, and 35 dpi. The three non-infected groups are not shown because *C. jejuni* was not detected in these groups at any sampling time point. Individual points represent individual chickens (*n* = 5 per group and time point), bars represent group means, and error bars indicate the standard error of the mean (SEM). Data are expressed as log_10_ CFU/g. Differences among dietary treatments were analyzed using two-way ANOVA followed by Tukey’s post hoc test. Asterisks indicate significant differences compared with the infected basal diet group (*p* < 0.05). ND = not detected.

**Figure 2:**
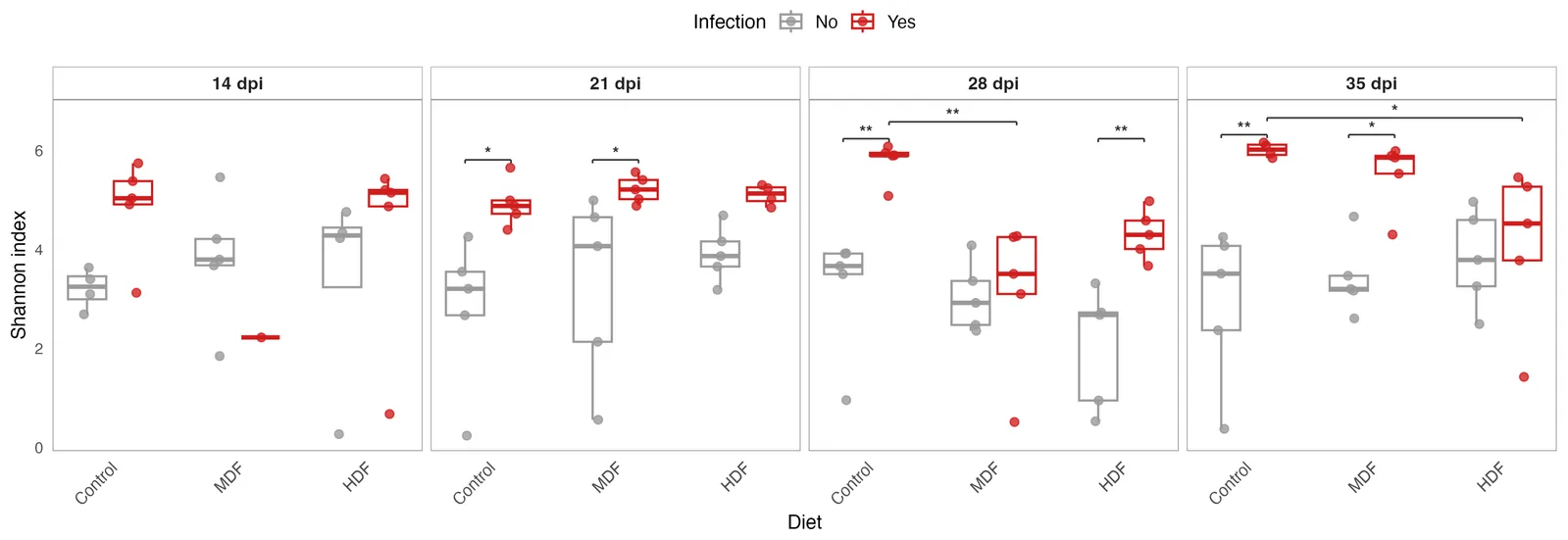
Effect of oat supplementation on cecal microbiota alpha-diversity in broiler chickens fed a basal diet, MDF, or HDF, with or without a *C. jejuni* infection. Alpha diversity was assessed using the Shannon diversity index. The boxplot shows the distribution of Shannon diversity values within each experimental group. The central line indicates the median, boxes represent the interquartile range (IQR), and whiskers indicate the data range. Color indicates infection status (gray, non-infected; red, infected). Asterisks indicate statistically significant pairwise differences between groups, with * *p* < 0.05, ** *p* < 0.01.

**Figure 3:**
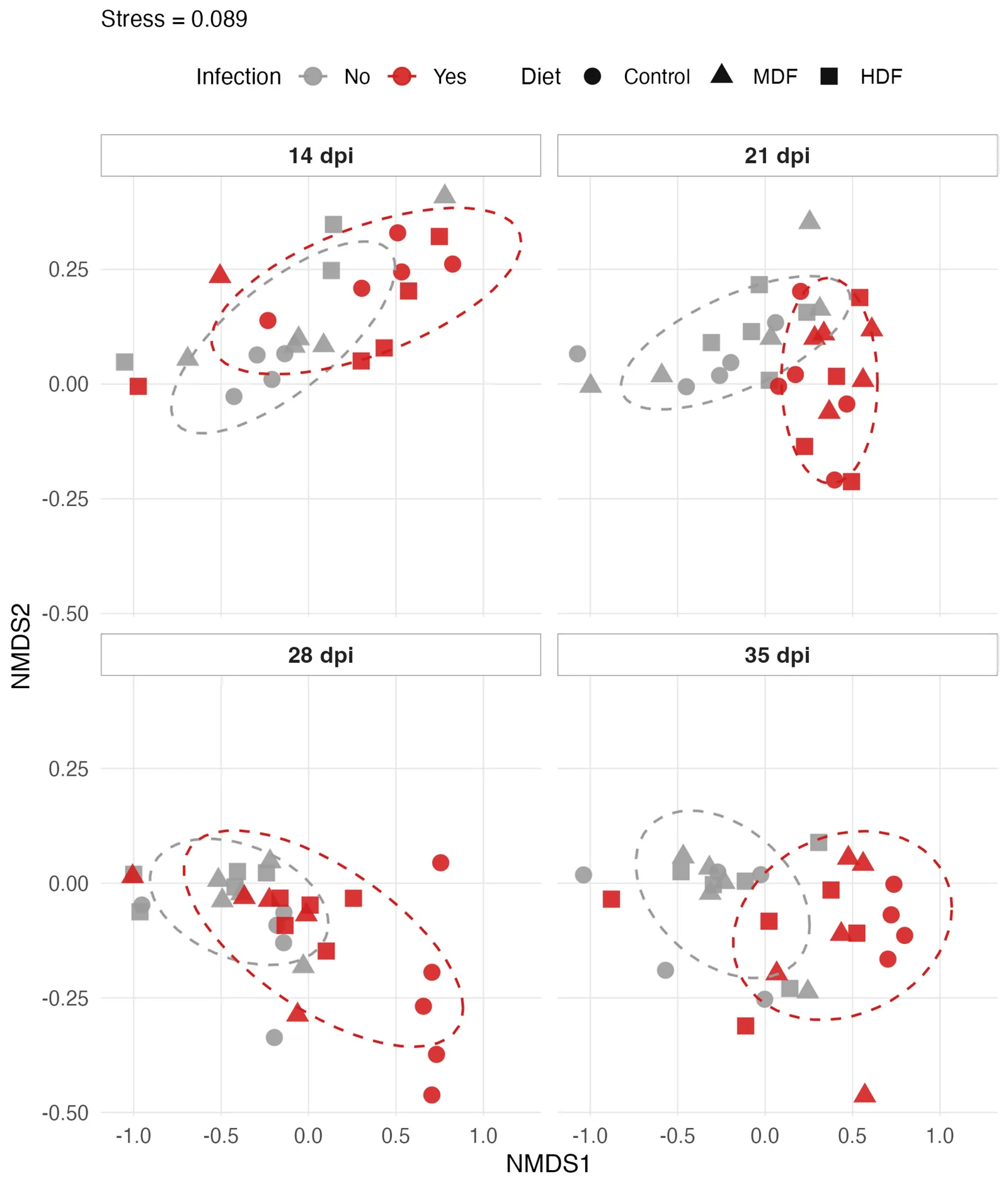
Non−metric multidimensional scaling (NMDS) ordination of cecal microbiota beta diversity in broiler chickens fed a basal diet, MDF, or HDF, with or without a *C. jejuni* infection across sampling time points. Ordinations were generated using Bray–Curtis dissimilarity distances. Clustering patterns reflect differences in microbial community structure among experimental groups. Color indicates infection status (gray, non-infected; red, infected), and symbol shapes indicate dietary treatment (circle, control, triangle, MDF, square, HDF): Dashed ellipses indicate the distribution of samples within the respective infection groups. Stress = 0.089.

**Figure 4:**
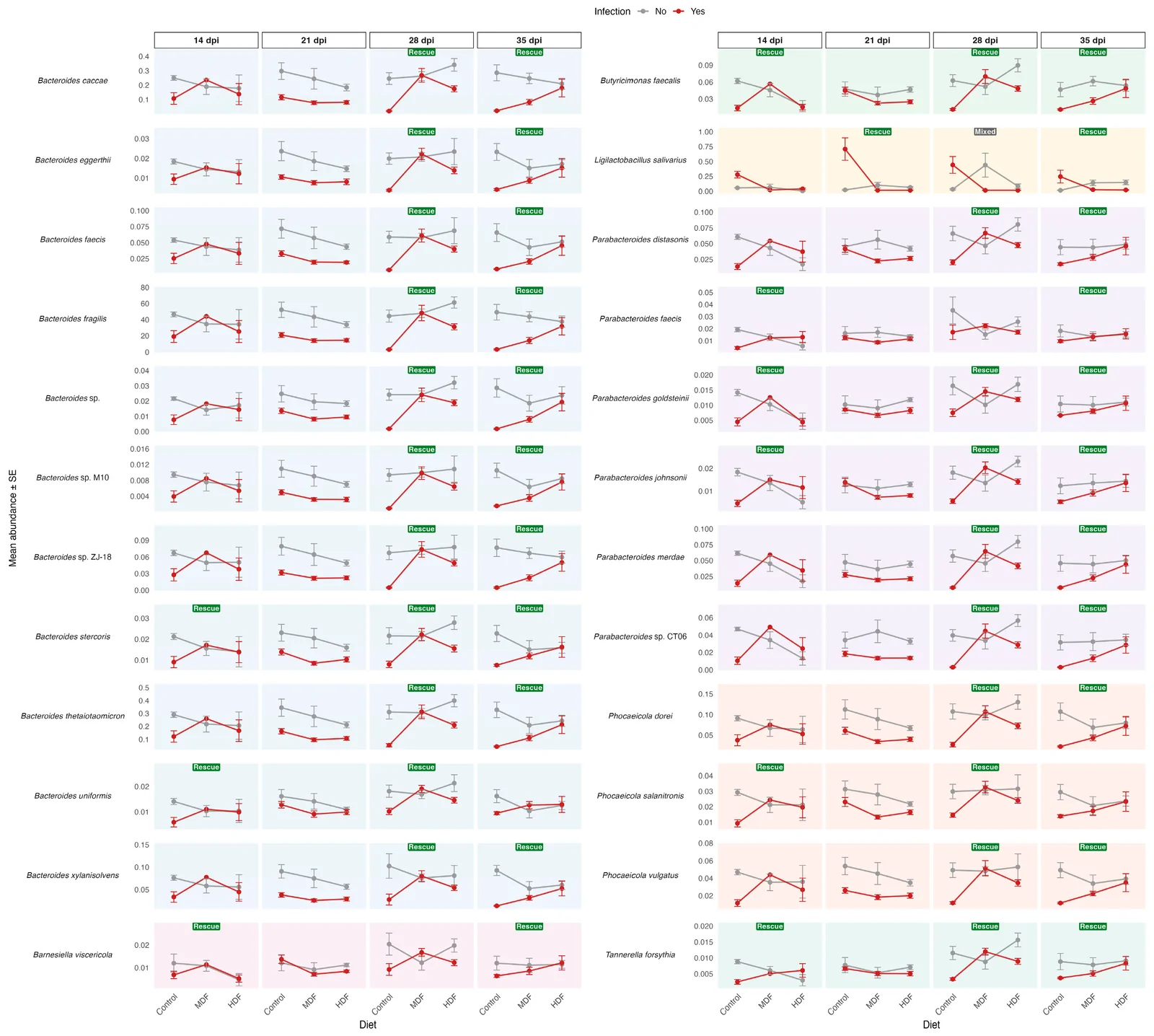
Mean relative abundance (±SE) of selected cecal bacterial species according to dietary treatment and *C. jejuni* infection status at 14, 21, 28, and 35 dpi. To improve readability, only bacterial species exhibiting a significant diet × infection interaction (*q* < 0.1) at two or more sampling time points are shown. Panels with a significant diet × infection interaction (*q* < 0.1) are labeled according to the interaction pattern (Rescue, Mixed, or Divergent), as defined in the methods.

**Figure 5:**
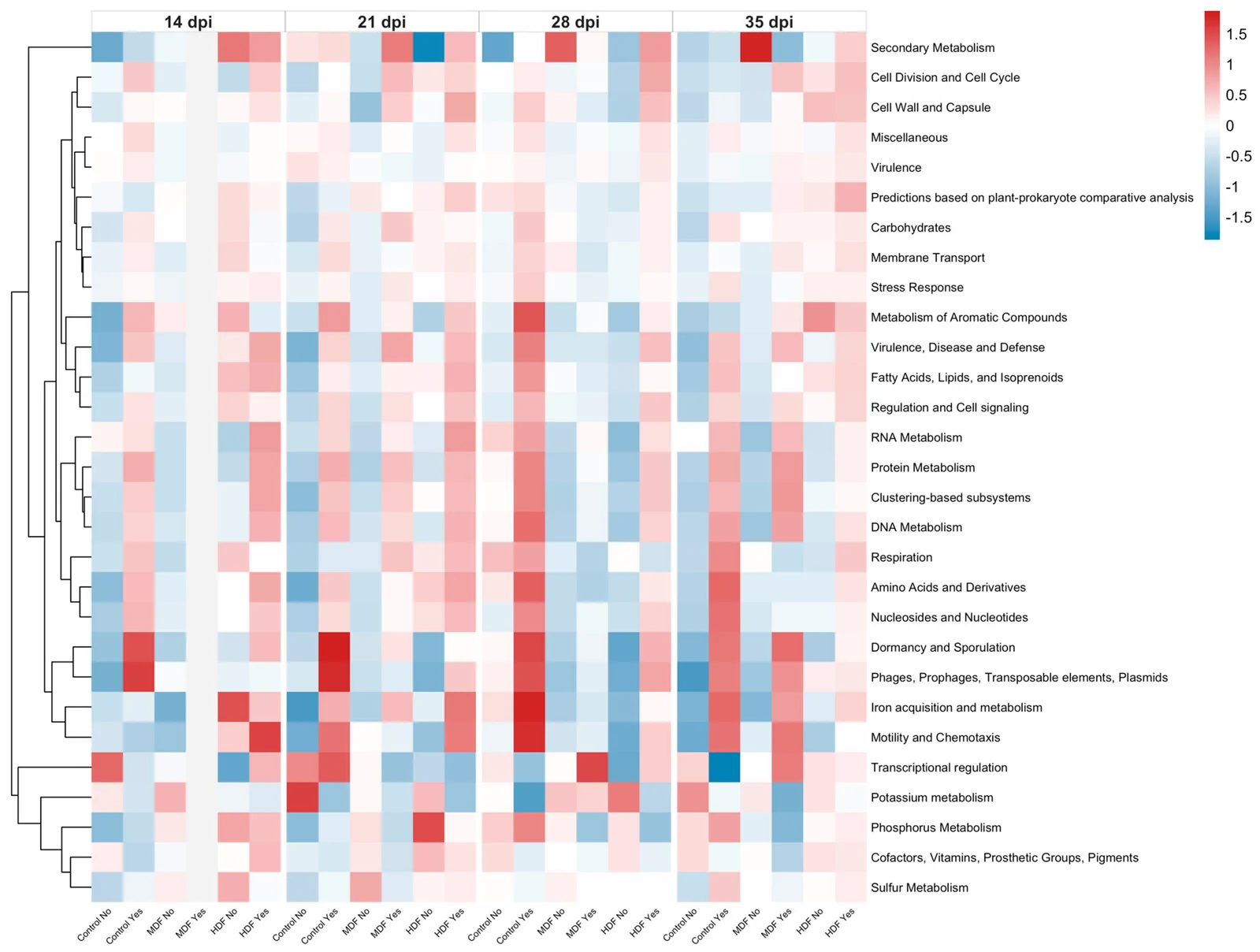
Predicted functional profiles of the cecal microbiome in broiler chickens fed a basal diet, MDF, or HDF, with or without *C. jejuni* infection. Heatmap showing descriptive, within-age standardized scores of SEED functional categories across sampling time points. Functional profiles were generated from shotgun metagenomic sequencing data using SEED subsystem annotations. Scores were standardized separately within each sampling age, with blue indicating lower and red indicating higher relative abundances compared with the other diet × infection groups at the same sampling age. Groups represented by fewer than four samples were excluded and are shown as blank cells.

**Figure 6:**
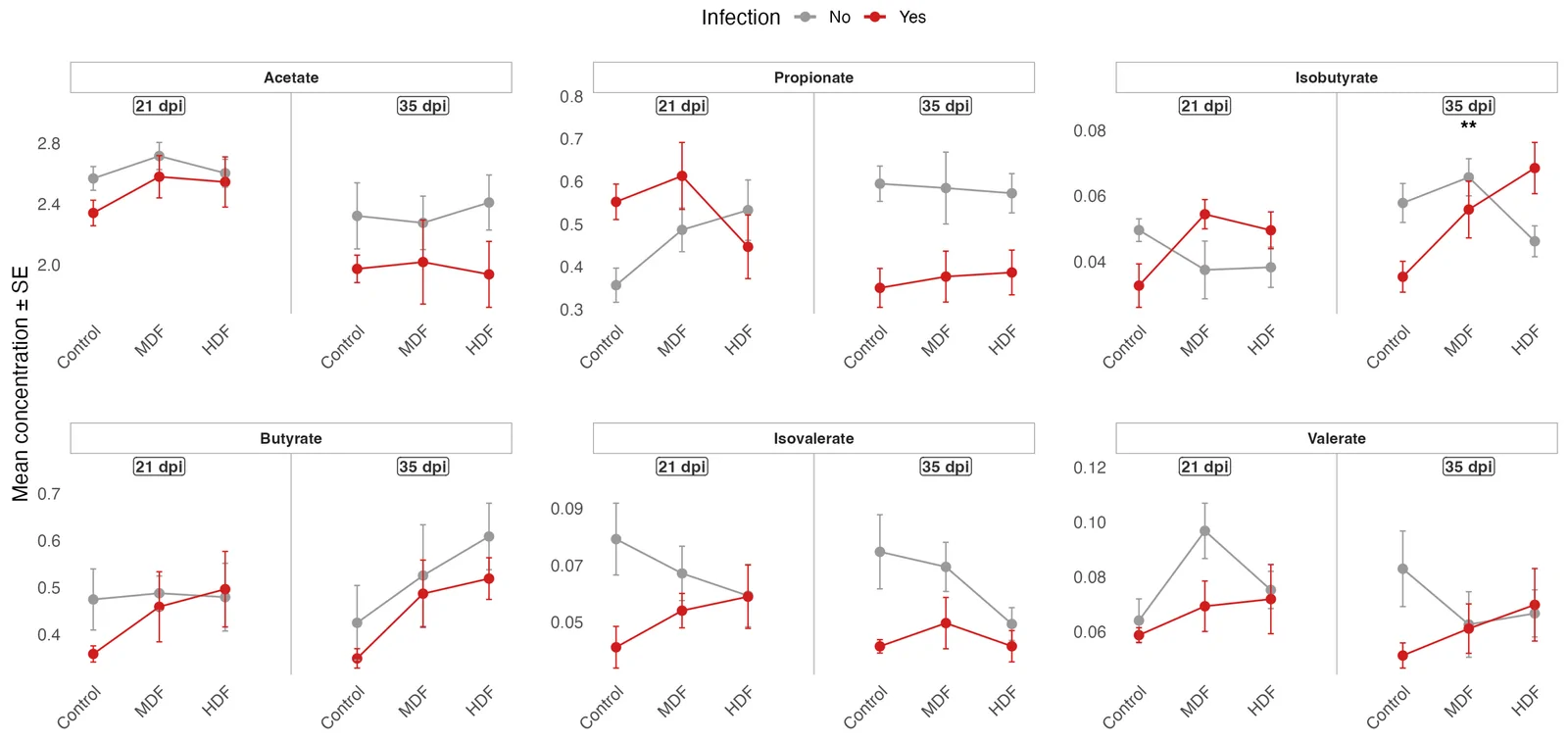
Cecal SCFA concentrations in broiler chickens fed a basal diet, MDF, or HDF, with or without *C. jejuni* infection, at 21 and 35 dpi. Data are presented as mean ± SE (*n* = 5). Diet × infection interactions were evaluated separately at each sampling time point using Type III two-way ANOVA. Asterisks indicate significant diet × infection interactions (** *p* < 0.01); panels without asterisks indicate no significant interaction.

**Figure 7:**
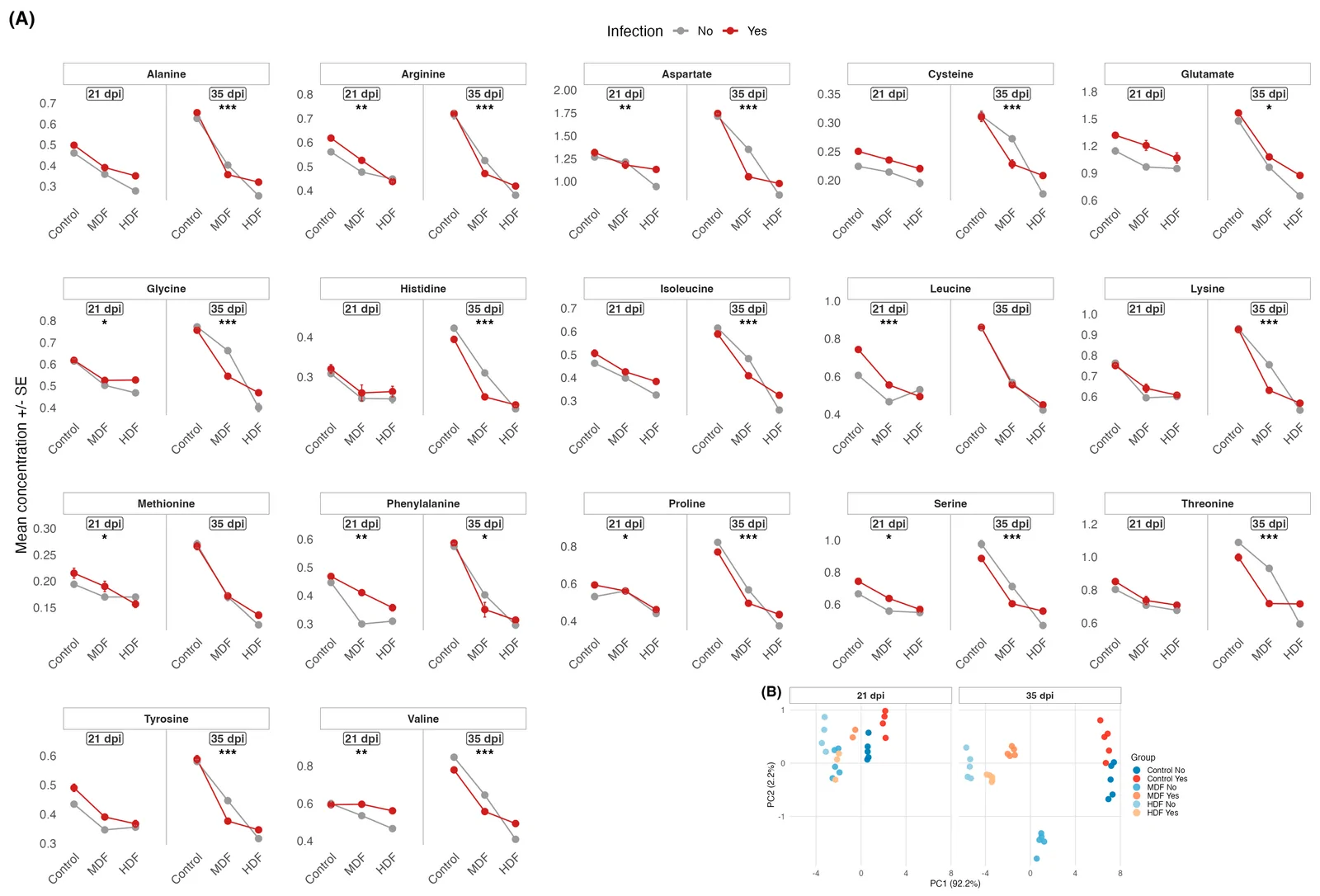
Effects of dietary treatment and *C. jejuni* infection on ileal amino acid concentrations and profiles at 21 and 35 dpi. (**A**) Ileal amino acid concentrations are presented as mean ± SE (*n* = 5) for broiler chickens fed a basal diet, MDF, or HDF, with or without *C. jejuni* infection. Diet × infection interactions were evaluated separately at each sampling time point using Type III two-way ANOVA. Asterisks indicate significant diet × infection interactions (* *p* < 0.05, ** *p* < 0.01, and *** *p* < 0.001). (**B**) Principal component analysis (PCA) of standardized ileal amino acid profiles showing clustering of the six diet × infection groups at each sampling time point.

**Table 1:** Ingredient composition, measured nutrient and amino acid profiles, and calculated AME_n_ values of the experimental diets across the starter, grower, and finisher phases.

	Starter			Grower			Finisher		
Ingredients g/kg	Basal	10% MDF	20% HDF	Basal	10% MDF	20% HDF	Basal	10% MDF	20% HDF
Corn	343.5			400			460	180.5	77.5
Wheat	100	375	253	100	440	319.5	100	300	300
Oat		100	200		100	200		100	200
Extracted soy	448	395	404	397	333	342	342	302	291
Sunflower oil	58	77	90	59	80	93	57	75	90
MCP	16	16	17	15	15	15	14.00	14.00	14.00
Limestone	18	18	18	15	15	15	14.00	14.00	13.00
Premix	5	5	5	5	5	5	5.00	5.00	5.00
Salt	3	3	3	3	3	3	3.00	3.00	3.00
Sodium bicarbonate	1	1	1	1	1	1	1	1	1
Methionine	4	4	4	3	3	3	3	2	2
Biolys (64.6%)	2	4	3	1	3	2	1	2	2
Threonine	1	1	1	1	1	1	0.5	1	1
Valine	0.5	1	1		1	0.5		0.5	0.5
Nutrient contents (%)									
DM	89.74	89.68	89.99	89.79	90.31	90.22	89.41	89.68	90.02
Crude protein	25.10	24.18	24.20	22.23	22.48	21.88	21.01	20.15	19.53
Crude fat	7.33	8.20	10.05	7.72	10.57	10.62	7.85	8.83	9.63
Crude fiber	4.61	5.13	6.66	4.48	6.50	6.50	4.04	4.97	5.83
Crude ash	7.31	7.31	7.32	6.85	7.01	6.86	6.58	6.44	6.17
Calcium	1.06	1.07	1.08	0.97	0.99	1.02	0.89	0.91	0.93
Phosphorus	0.84	0.88	0.89	0.74	0.81	0.72	0.69	0.69	0.62
Starch	32.67	29.72	26.58	35.42	29.09	28.31	36.79	34.23	32.98
NDF	13.72	15.36	16.98	13.87	17.11	17.18	14.00	15.60	17.24
IDF	13.20	13.39	16.28	12.46	15.23	15.92	10.98	12.21	15.62
SDF	4.29	4.92	5.20	4.19	4.68	5.23	4.26	4.38	4.86
Arabinoxylan	3.54	3.71	3.32	3.72	3.93	3.56	3.95	4.06	3.94
Soluble arabinoxylan	0.15	0.29	0.26	0.13	0.31	0.28	0.17	0.25	0.29
Beta-glucan	0.29	0.91	1.35	0.26	0.91	1.41	0.3	0.88	1.33
Soluble bata-glucan	0.05	0.42	0.72	0.06	0.42	0.75	0.08	0.40	0.70
CYS	0.38	0.38	0.37	0.34	0.36	0.35	0.32	0.33	0.33
ASP	2.60	2.40	2.50	2.27	2.24	2.20	2.12	1.95	1.92
MET	0.71	0.70	0.72	0.59	0.60	0.59	0.54	0.49	0.48
THR	1.02	0.95	0.95	0.91	0.89	0.87	0.82	0.83	0.80
SER	1.22	1.17	1.18	1.09	1.07	1.06	1.03	0.98	0.95
GLU	4.63	4.81	4.69	4.11	4.53	4.41	3.94	4.05	3.88
PRO	1.36	1.40	1.34	1.25	1.28	1.26	1.21	1.21	1.15
GLY	1.01	0.99	1.00	0.90	0.92	0.91	0.84	0.82	0.79
ALA	1.12	0.99	1.01	1.02	0.93	0.90	1.01	0.86	0.83
VAL	1.18	1.20	1.22	1.01	1.10	1.05	0.97	0.95	0.96
ILE	1.05	1.00	1.01	0.93	0.93	0.91	0.87	0.85	0.80
LEU	1.95	1.72	1.74	1.78	1.62	1.59	1.71	1.52	1.44
TYR	0.83	0.77	0.79	0.73	0.71	0.71	0.69	0.65	0.63
PHE	1.22	1.18	1.17	1.09	1.12	1.06	1.03	0.99	0.94
HIS	0.65	0.58	0.58	0.58	0.56	0.54	0.55	0.49	0.50
LYS	1.50	1.51	1.47	1.25	1.26	1.25	1.15	1.14	1.11
ARG	1.67	1.62	1.61	1.48	1.51	1.39	1.35	1.27	1.31
AMEn (MJ/kg)	12.65	12.65	12.65	13.00	13.00	13.00	13.2	13.2	13.2

## Data Availability

The raw sequencing data generated in this study have been deposited in the NCBI Sequence Read Archive (SRA) under BioProject accession number PRJNA1471692 (Submission ID: SUB16216877). The data are available through the NCBI BioProject database (http://www.ncbi.nlm.nih.gov/bioproject/1471692) (accessed on 21 September 2026) and will be publicly accessible upon publication of this article.
